# Comparison of Normal and Pre-Eclamptic Placental Gene Expression: A Systematic Review with Meta-Analysis

**DOI:** 10.1371/journal.pone.0161504

**Published:** 2016-08-25

**Authors:** O. Brew, M. H. F. Sullivan, A. Woodman

**Affiliations:** 1 University of West London, Brentford, Middlesex, United Kingdom; 2 Institute of Reproductive & Developmental Biology, Imperial College London, Hammersmith Hospital Campus, London, United Kingdom; 3 University of West London, Ealing, London, United Kingdom; Utah State University, UNITED STATES

## Abstract

Pre-eclampsia (PE) is a serious multi-factorial disorder of human pregnancy. It is associated with changes in the expression of placental genes. Recent transcription profiling of placental genes with microarray analyses have offered better opportunities to define the molecular pathology of this disorder. However, the extent to which placental gene expression changes in PE is not fully understood. We conducted a systematic review of published PE and normal pregnancy (NP) control placental RNA microarrays to describe the similarities and differences between NP and PE placental gene expression, and examined how these differences could contribute to the molecular pathology of the disease. A total of 167 microarray samples were available for meta-analysis. We found the expression pattern of one group of genes was the same in PE and NP. The review also identified a set of genes (PE unique genes) including a subset, that were significantly (p < 0.05) down-regulated in pre-eclamptic placentae only. Using class prediction analysis, we further identified the expression of 88 genes that were highly associated with PE (p < 0.05), 10 of which (LEP, HTRA4, SPAG4, LHB, TREM1, FSTL3, CGB, INHA, PROCR, and LTF) were significant at p < 0.001. Our review also suggested that about 30% of genes currently being investigated as possibly of importance in PE placenta were not consistently and significantly affected in the PE placentae. We recommend further work to confirm the roles of the PE unique and associated genes, currently not being investigated in the molecular pathology of the disease.

## Introduction

Pre-eclampsia (PE), a major cause of perinatal mortality complicates up to 8% of all pregnancies in Western countries [1–3]. It is one of the top 4 causes of maternal mortality and morbidity worldwide, causing 10 to 15% of maternal deaths [2–4]. PE is characterised by new hypertension (blood pressure of ≥140/90 mmHg) on two separate readings at least 6 hours apart presenting after 20 weeks' gestation in conjunction with clinically relevant proteinuria (≥300mg) per 24 hours [5].

PE is a multifactorial disease, and while there is a cautious acceptance of links between familial concordance and maternal polymorphism in the pathogenesis of the disease [6–13], the placenta is suggested as the primary cause of PE [14,15], Nonetheless, there is a degree of uncertainty, especially about the roles of gene regulation and expression in the molecular pathogenesis of the disease. Expectedly, knowledge on placental gene expression is advancing [16–18]. And while recent meta-analysis of Relative Gene Expression (RGE) in NP and PE placentae have linked the changes in specific genes in the placenta to PE [13,19], these studies have often focused on identifying genes that are either highly up-regulated or down-regulated between the case and control matched samples. Traditionally, this approach is suggested as highly suitable for candidate gene discovery or class prediction studies [20–22]. However, this methodology is lately suggested as less sensitive for microarray studies that seek to account for variability in gene expression across sample within same class or to map the molecular pathology of a disease from 'noisy' data sets [23–26]. We therefore examined whether RGE analysis would identify same PE genes as Absolute Gene Expression (AGE) analysis, and also to determine the functional roles of gene sets or families that are equally expressed at high or low levels in both NP and PE placentae.

Therefore, in this study we provide evidence that AGE analyses identify gene sets whose combined expression patterns could uniquely characterise biological and functional phenotype for PE placentae. We further provide evidence for putative inter-relationships and contributory roles of equally low or high level expressed genes in the molecular pathology of PE.

## Materials and Methods

### Study selection

Public data repositories Gene Expression Omnibus (GEO) and ArrayExpress Archive were systematically searched in accordance with PRISMA and MIAME in December 2014, and repeated in June 2015. No time limit for data publication was set. Search terms used were NP placenta, PE and Term placenta explant. Study series with no report on placental tissue but other tissues such as Chorionic villous tissue, Decidua, Trophoblast cell lines and Basement membrane were excluded. Similarly, study series with no matched control group; control group composed of pregnancies complicated by small for gestational age fetuses; gestational diabetes, Non-homo sapiens control; and Non-term placentae were excluded. Also, duplicate samples; Methylation profiling array; Protein profiling array; Long non-coding RNAs (long ncRNAs, lncRNA); and all complications of human pregnancy other than PE were excluded.

### Array Processing and Quality Control

Data for each sample included were downloaded from GEO (or from ArrayExpress if not available in GEO). The series data were prepared according to INMEX [27] requirement for meta-analysis, and exported into INMEX. Probe IDs from the different platforms were re-annotated in INMEX using the November, 2012 annotation information obtained from the NCBI GenBank and Bioconductor into Entrez gene IDs. Multiple probes mapping to the same gene were presented as an average for combined probes and thence referred to as genes.

To prepare the data for differential expression analysis using Linear Model for Microarrays (Limma), the data was log transformed into additive scale, and then quantile normalised. Microarray quality appraisal was further performed using INMEX built-in protocol. Firstly, study series with low quality samples was defined as samples with >60% missing data, and were rejected. Using the INMEX inbuilt re-annotation protocol, study series with less than 10 common genes were also excluded from further analysis.

### Finding Significantly Expressed Genes in Pre-eclampsia

Significantly expressed gene was defined as a gene that shows consistent stronger aggregated differential expression (DE) profile across the multiple datasets [24,27]. Therefore the DE genes were identified by combining P-values from the multiple studies using Fisher's method (-2*∑Log(p)) (p<0.05) for Relative expressions or RankProduct analysis for Absolute expressions.

RankProduct analysis, a non-parametric statistic [24], was used to identify genes that were consistently up-regulated or down-regulated in PE or NP placentae. The RankProduct analysis combined the gene rank from the different arrays together instead of using actual expression data to select genes that were consistently ranked high or low [24,26]. The product ranks from all samples were then calculated as the test statistic in100X permutations with False Discovery Rate (FDR) Confidence at 1 –alpha = 95.0%. Genes with consistently high ranks (smaller rank product) across the different microarrays were classified as up-regulated. Genes with consistently low ranks (larger rank products) across the different arrays at the stated FDR were classified as down-regulated, whereas genes with inconsistent rank product across the different studies were classified as non-significant [28].

### Gene-Disease Association Analysis

Gene-Disease Association analysis was performed to identify genes whose expression could be associated with PE placentae [29]. Using BRBArray Tools five prediction methods were performed: Compound covariate predictor, Diagonal linear discriminant analysis, K-nearest neighbours (for K = 1 and 3), Nearest Centroid, and Support vector machines. With a fixed internal random seed, genes were selected using a combination of univariate F-test (p < 0.05), and Leave-one-out cross-validation at 100 permutations, and further evaluated with ROC curve analysis.

### Functional Role Assignment

Biological relevance of the differentially expressed genes was determined using gene enrichment analysis programmes in WebGestalt [30]. Briefly, Kyoto Encyclopedia of Genes and Genomes (KEGG) Homo sapiens genome pathways database [31] was probed with the differentially expressed genes to identify statistically enriched pathways. The Benjamini & Hochberg (BH) hypergeometric test [32] was used for all enrichment evaluation analyses, with adjusted p values based on R function p.adjust.

## Results

**Fig 1:**
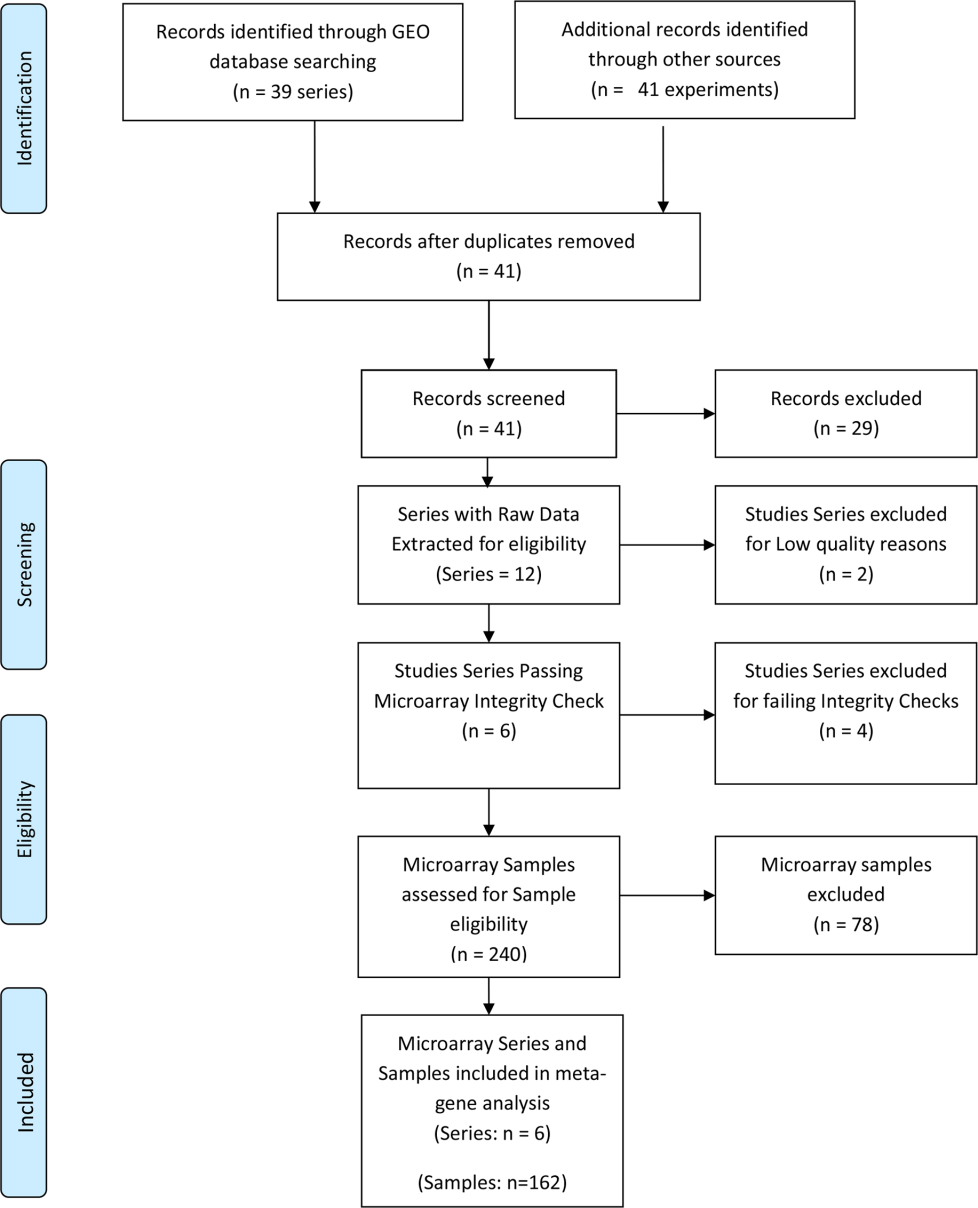
PRISMA flow chart of NP and PE Gene Expression Systematic Review.

Following the systematic search, a total of 41 microarray study series were identified (Fig 1). Twelve of the study series met the inclusion criteria, and 29 series (S1 Table) were excluded based on the eligibility criteria. Of the 12 series that met the eligibility criteria, 6 series (GSE30186, GSE25906, GSE35574, GSE43942, GSE4707, GSE47187) (Table 1) passed the quality and integrity checks. The remaining six failed the INMEX microarray quality and integrity assessments and were further excluded (S2 Table). Altogether 167 samples, consisting of 68 PE and 99 NP met the sample inclusion criteria for the meta-analysis. A total of 16701 genes passed filtering criteria.

**Table 1:** Profile of Microarray Series included in Pre-eclampsia Meta-gene Analysis.

GEO Accession	Type	Organism	Assays included	Platform	Release Date
GSE47187	transcription profiling by array	Homo sapiens	10	GPL14550	01/10/2013
GSE43942	transcription profiling by array	Homo sapiens	12	GPL10191	01/02/2013
GSE30186	transcription profiling by array	Homo sapiens	12	GPL10558	24/06/2011
GSE25906	transcription profiling by array	Homo sapiens	60	GPL6102	10/12/2010
GSE4707	transcription profiling by array	Homo sapiens	14	GPL1708	07/05/2008
GSE35574	transcription profiling by array	Homo sapiens	59 (IUGR samples excluded)	GPL6102	07/02/2012

### Patterns of Gene Expression in NP and PE Placentae

The samples were analysed to determine the patterns of gene expression in NP and PE placentae. We used AGE and RGE analyses to characterise the respective patterns in PE and NP placentae.

#### AGE analysis for NP and PE placental genes

RankProd meta-analysis was used to identify AGE in NP or PE (Table 2). Significant AGE was defined as genes whose product of expression were persistently ranked as positively (up) or negatively (down)-regulated across all PE only or NP only placentae, at a given false discovery rate (FDR<0.05). Data output was expressed as rank product of mean expression levels. For NP, a total of 1922 genes were identified as consistently significant (FDR < 0.05). Of these, 846 genes were negatively regulated and 1076 were positively regulated (Table 2). The expression levels of 14779 genes in NP placentae were inconsistent and were classified as non-significant. In contrast, the expression of 9540 genes in PE placentae was consistent and significant (FDR < 0.05) (Table 2). Of these, 5146 (54%) genes were significantly down-regulated and 4394 (46%) genes were up-regulated in the PE placentae. The expression levels of 7161 genes in PE placentae were inconsistent and thus were classified as non-significant (Table 2).

**Table 2:** Differentially Expressed Genes in NP and PE Placentae.

		Absolute PE only	Absolute NP only	Relative PE/NP
Negatively Significant Genes	*#* of Negatively Significant Genes	5146	846	2197
	% of Negatively Significant Genes	31%	5%	13%
Positively Significant Genes	*#* of Positively Significant Genes	4394	1076	2152
	% of Positively Significant Genes	26%	6%	13%
Non-Significant Genes	*#* of Non-Significant Genes	7161	14779	12352
	% of Non-Significant Genes	43%	88%	74%

A total of 167 microarray samples (PE = 68; NP = 99) were meta-analysed as case-to-control matched samples with Fisher’s method or as case (PE) only and control (NP) only with RankProd analysis. About 31% more genes were identified as differentially expressed (DE) in PE only than in case matched baseline (NP) subtraction PE. RankProd analysis Confidence at (1—alpha): 95.0%; False Significant Proportion: 0.05 or less; p value threshold for fisher’s metaP = 0.05. PE = Pre-eclampsia Placentae; NP = Normal Placentae.

#### RGE analysis for PE placental genes

**Fig 2:**
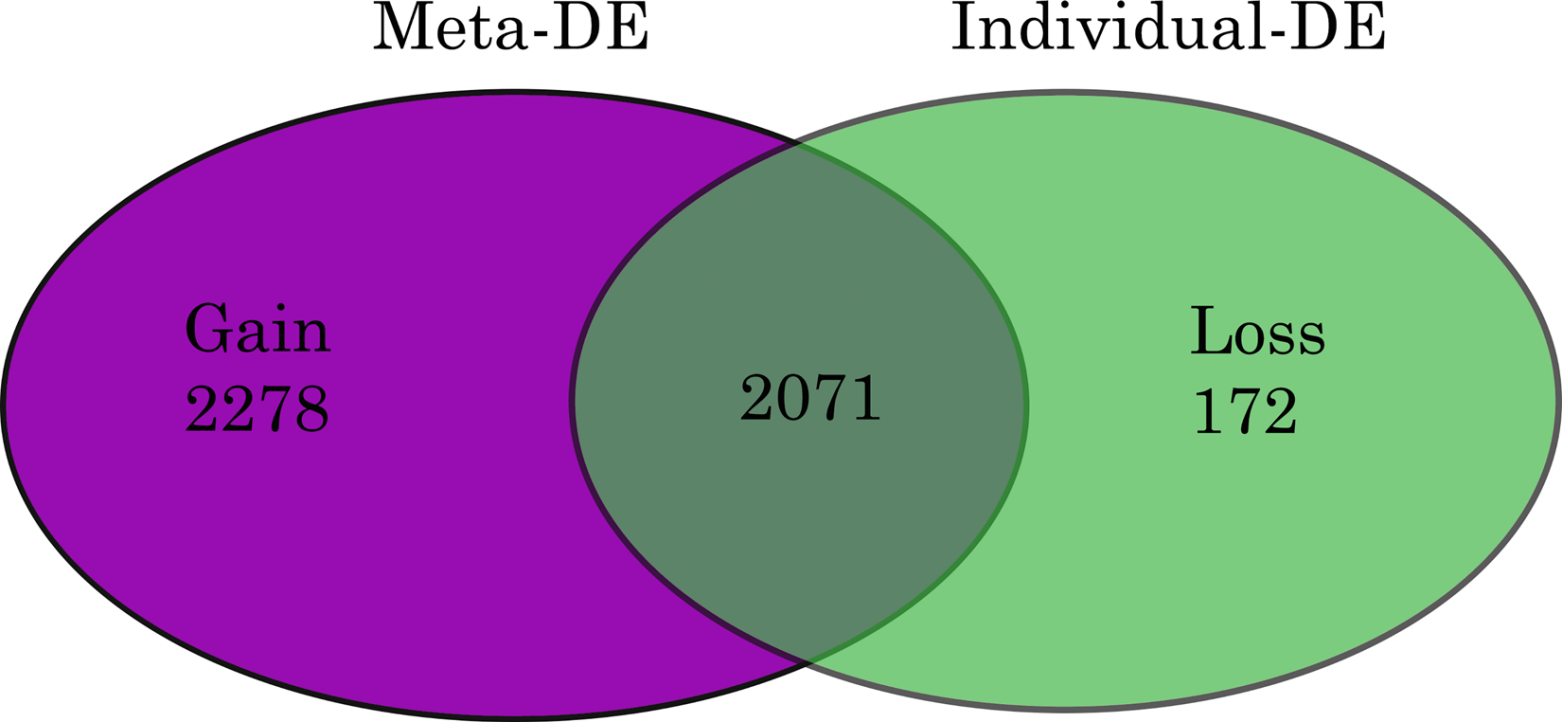
Venn Diagram of Differentially Expressed Genes in Pre-eclampsia. Fig shows differentially expressed (DE) genes in PE. Meta-analysis of ‘p’ values was performed using Fishers method. MetaDE = differentially expressed genes following metaP-analysis. Gain = DE gene only found in meta-analysis result but not in any individual analysis. Loss = DE genes identified in any individual analysis, but not in the meta-analysis. Gain and Loss genes were calculated by comparing DE genes identified by meta-analysis to those from analysing individual datasets.

RGE was defined as the relative quantitation of the differences in the expression level of a gene between the PE and NP placental samples [27]. The data output was expressed as a fold-change of expression levels in PE relative to NP. Using fisher’s method to combine p values, the expressions of 4349 genes were identified as significant in PE (p <0.05), relative to NP (Table 2). Of these, 2197 (13%) genes were negatively regulated, and 2152 (13%) were positively regulated (Table 2). Fig 2 shows that 2071 of these genes were differentially expressed across the study series before meta-analysis, and a further 2278 genes were significant (p <0.05) only after meta-analysis. The expression of 172 other genes lost significance after meta-analysis.

#### Trends in placental gene expression and PE unique genes

**Fig 3:**
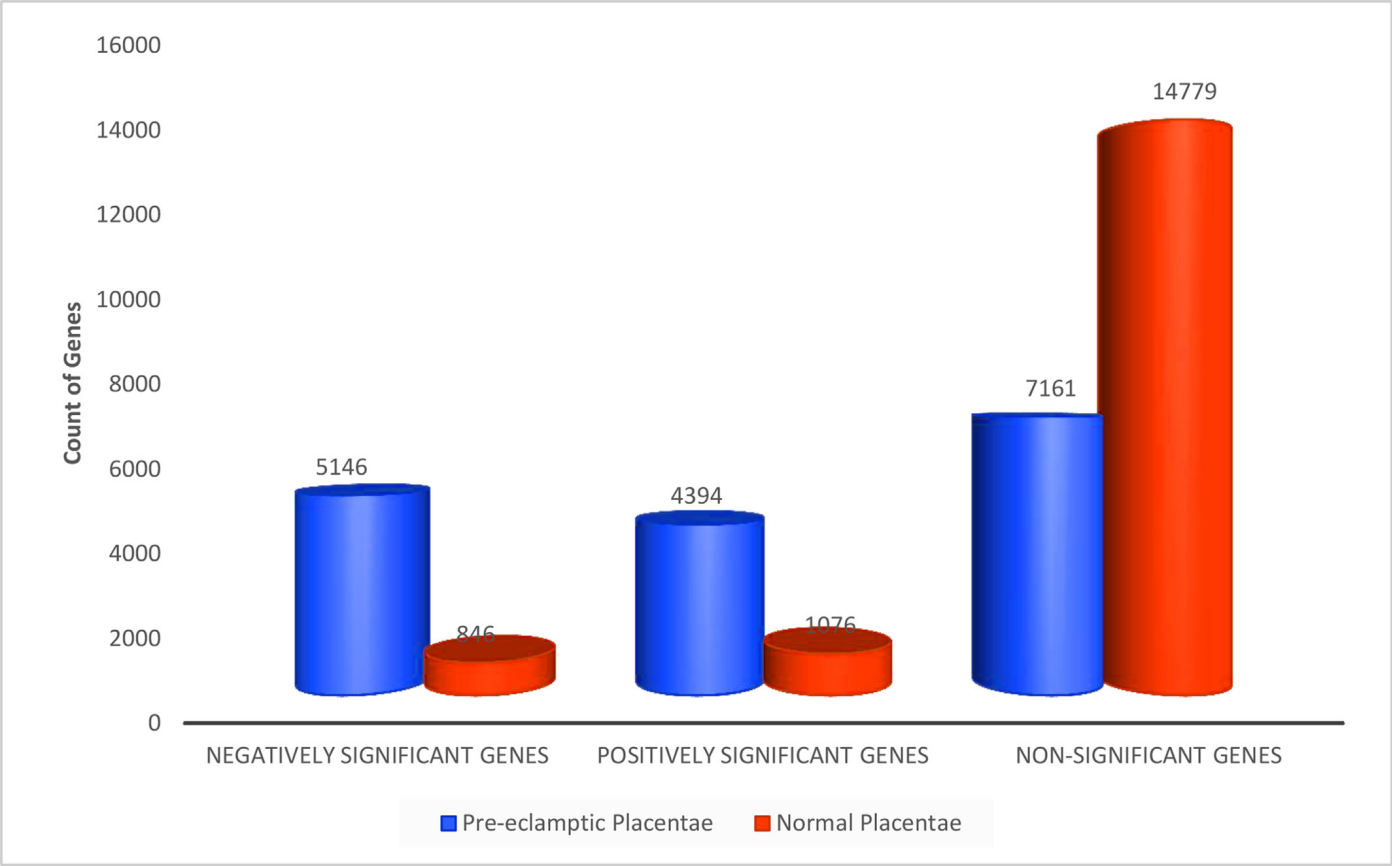
Absolute Gene Expression in Normal and Pre-eclamptic Placentae. Counts of genes significantly regulated in PE and NP placentae. Gene ratios for PE:NP are 6:1, 4:1 and 1:2 respectively for negatively significant, positively significant and non-significant genes. Genes identified with Rankprod statistics in100X permutations (FDR Confidence at 1 –alpha = 95.0%).

Trends in the changes to PE placental gene expression were determined by examining the relationships between PE and NP Absolute and Relative gene sets. First, we compared the gene counts in the respective gene sets from the Absolute PE and NP analyses. Fig 3 shows a 6 fold increase in the number of negative significant genes in PE than in NP. Similarly, there was a 4 fold increase in positive significant genes in PE than in NP (Fig 3). The proportion of non-significant genes in NP following AGE was twice the concentration found in PE non-significant gene set.

Interestingly, while the proportions of genes identified as positive or negative significant from RGE were twice less than those identified from AGE (Table 2), the number of the Relative PE non-significant genes was similar to the Absolute NP non-significant genes. We therefore examined further, whether there was any relationship between the Absolute NP non-significant genes, Absolute PE significant and Relative PE significant genes.

**Fig 4:**
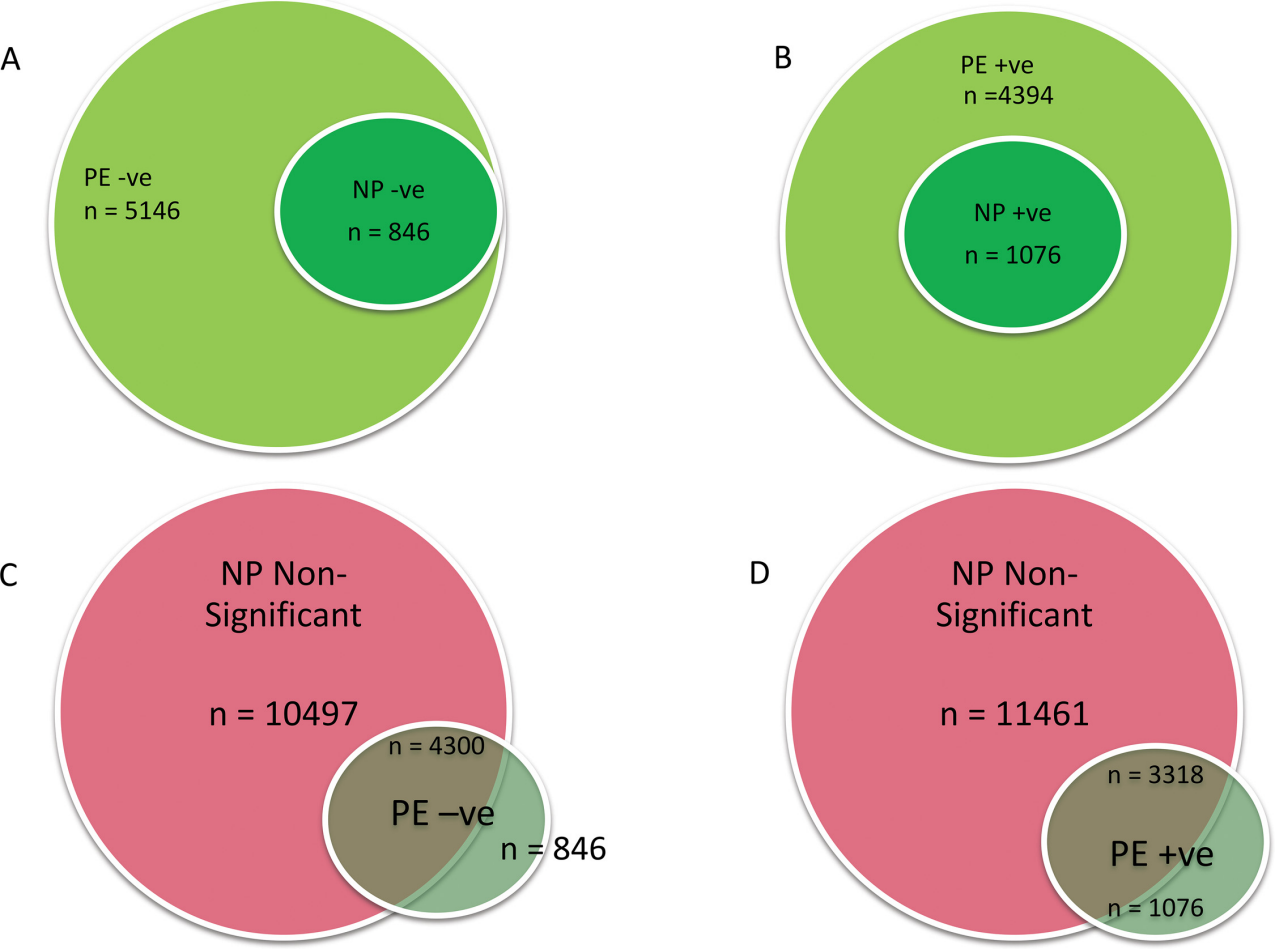
Relation between NP and PE Placental Gene Expression. Significantly down-regulated (a) or up-regulated (b) genes in PE were compared with down (n = 864) or up-regulated (n = 1076) genes in NP respectively. All genes significantly up or down regulated in NP were respectively regulated in PE. In c & d, non-significantly differentiated genes in NP were compared with PE up or down-regulated. PE = pre-eclamptic placenta; NP = normal placenta; -ve = down-regulated; +ve = up-regulated

Using BioVenn [33] we identified four sets of genes. First, the comparison of the PE and NP Absolute genes (Fig 4A & 4B) showed 2 sets of down-regulated gene: (1) a set of genes that were significantly (p< 0.05) down-regulated in both NP and PE (n = 846; Fig 4A; S4 Table); (2) a second set of genes that were significantly (p< 0.05) down-regulated only in PE placentae (n = 4300; p< 0.05; S5 Table). The third set of genes consisted of a group of 1076 genes significantly (p<0.05) up-regulated in both NP and PE (S6 Table). The fourth set was a group of 3318 genes, that were significantly (p< 0.05) up-regulated only in PE (S7 Table).

Further comparison of the PE negative and positive significant gene sets with NP non-significant gene sub-group showed that all the PE unique genes were not significantly regulated in NP placenta (Fig 4C & 4D). Altogether, there were 7618 more significantly regulated genes in PE than were in NP at the FDR Confidence of 1 –alpha = 95.0%.

### Links between Relative and Absolute PE Significant Genes

We further examined the relationship between the PE significant Relative and Absolute genes. All 4394 PE Absolute positive (up-regulated) significant genes were compared with the 2152 PE Relative positive significant genes. We expected all Relative PE genes to be identified amidst the Absolute PE gene sets. However, only 79% (n = 1688) of the Relative positive significant genes were identified in the PE Absolute positive genes. A much smaller number (24%, n = 524) of the total PE Relative negative (down-regulated) significant genes (n = 2197) were identified in the Absolute PE negative significant genes. Overall, only 51% of the Relative significant genes were identified in the Absolute gene sets, with majority localised within the positive significant gene set. Further examination showed that the expression signals of the significant Relative genes unmatched to Absolute genes were previously classified as inconsistent and non-significant by the AGE analysis. In contrast, the Absolute genes not matched to Relative genes typically showed low level expression profile or were similarly expressed in both NP and PE placentae.

### PE Placental Associated (PPA) Genes and Current Research

We tested the hypothesis that NP and PE placental gene expression profiles do not differ, and that a prediction analysis would not discriminate between the NP and PE genes but only pick up the random noise in the data set. To examine this, all 16701 genes from 99 NP placentae and 68 PE placental microarrays samples were tested and the expression of 88 genes (Table 3) was significantly (p <0.05) associated with PE placentae (Pre-eclamptic Placenta Associated, PPA).

**Table 3:** Pre-eclamptic Placental Associated Genes.

Symbol	Name	EntrezID	Accession	P Value
LEP	leptin	3952	NM_000230	0.0003
HTRA4	HtrA serine peptidase 4	203100	NM_153692	0.0009
SPAG4	sperm associated antigen 4	6676	NM_003116	0.003
LHB	luteinizing hormone beta polypeptide	3972	NM_000894	0.003
TREM1	triggering receptor expressed on myeloid cells 1	54210	NM_001242589	0.005
FSTL3	follistatin-like 3 (secreted glycoprotein)	10272	NM_005860	0.005
CGB	chorionic gonadotropin, beta polypeptide	1082	NM_000737	0.005
INHA	inhibin, alpha	3623	NM_002191	0.006
PROCR	protein C receptor, endothelial	10544	NM_006404	0.007
LTF	lactotransferrin	4057	NM_001199149	0.008
FLT1	fms-related tyrosine kinase 1	2321	NM_001159920	0.011
CORO2A	coronin, actin binding protein, 2A	7464	NM_003389	0.011
S100A14	S100 calcium binding protein A14	57402	NM_020672	0.012
LPL	lipoprotein lipase	4023	NM_000237	0.012
GP6	glycoprotein VI (platelet)	51206	NM_001083899	0.012
SIGLEC6	sialic acid binding Ig-like lectin 6	946	NM_001177547	0.013
ZNF114	zinc finger protein 114	163071	NM_153608	0.017
BHLHE40	basic helix-loop-helix family, member e40	8553	NM_003670	0.017
EPS8L1	EPS8-like 1	54869	NM_017729	0.018
QPCT	glutaminyl-peptide cyclotransferase	25797	NM_012413	0.018
BTNL9	butyrophilin-like 9	153579	NM_152547	0.019
PLIN2	perilipin 2	123	NM_001122	0.019
NTRK2	neurotrophic tyrosine kinase, receptor, type 2	4915	NM_001007097	0.019
KRT15	keratin 15	3866	NM_002275	0.019
NEK11	NIMA-related kinase 11	79858	NM_001146003	0.019
PAPPA2	pappalysin 2	60676	NM_020318	0.019
BCL6	B-cell CLL/lymphoma 6	604	NM_001130845	0.020
SAPCD2	suppressor APC domain containing 2	89958	NM_178448	0.020
ENG	endoglin	2022	NM_000118	0.020
HK2	hexokinase 2	3099	NM_000189	0.020
NDRG1	N-myc downstream regulated 1	10397	NM_001135242	0.021
GBA	glucosidase, beta, acid	2629	NM_000157	0.022
CYP2J2	cytochrome P450, family 2, subfamily J, polypeptide 2	1573	NM_000775	0.022
PLA2G16	phospholipase A2, group XVI	11145	NM_001128203	0.023
SLC11A1	solute carrier family 11 (proton-coupled divalent metal ion transporter), member 1	6556	NM_000578	0.023
NPNT	nephronectin	255743	NM_001033047	0.023
MS4A15	membrane-spanning 4-domains, subfamily A, member 15	219995	NM_001098835	0.023
HILPDA	hypoxia inducible lipid droplet-associated	29923	NM_001098786	0.023
GPT2	glutamic pyruvate transaminase (alanine aminotransferase) 2	84706	NM_001142466	0.024
GREM2	gremlin 2, DAN family BMP antagonist	64388	NM_022469	0.024
TMEM178A	transmembrane protein 178A	130733	NM_001167959	0.024
RASEF	RAS and EF-hand domain containing	158158	NM_152573	0.024
LRG1	leucine-rich alpha-2-glycoprotein 1	116844	NM_052972	0.025
ERO1L	ERO1-like (S. cerevisiae)	30001	NM_014584	0.025
SLC16A3	solute carrier family 16 (monocarboxylate transporter), member 3	9123	NM_001042422	0.026
SH3BP5	SH3-domain binding protein 5 (BTK-associated)	9467	NM_001018009	0.027
PPL	periplakin	5493	NM_002705	0.027
ULBP1	UL16 binding protein 1	80329	NM_025218	0.028
FBXL16	F-box and leucine-rich repeat protein 16	146330	NM_153350	0.029
FCRLB	Fc receptor-like B	127943	NM_001002901	0.029
SLC6A8	solute carrier family 6 (neurotransmitter transporter), member 8	6535	NM_001142805	0.031
CCR7	chemokine (C-C motif) receptor 7	1236	NM_001838	0.033
SFN	stratifin	2810	NM_006142	0.034
MID1	midline 1 (Opitz/BBB syndrome)	4281	NM_000381	0.034
GLIS3	GLIS family zinc finger 3	169792	NM_001042413	0.034
STBD1	starch binding domain 1	8987	NM_003943	0.035
TNFAIP2	tumor necrosis factor, alpha-induced protein 2	7127	NM_006291	0.036
DSCR4	Down syndrome critical region gene 4	10281	NM_005867	0.037
MTSS1L	metastasis suppressor 1-like	92154	NM_138383	0.038
TPBG	trophoblast glycoprotein	7162	NM_001166392	0.038
KIAA1919	KIAA1919	91749	NM_153369	0.038
EBI3	Epstein-Barr virus induced 3	10148	NM_005755	0.038
CLC	Charcot-Leyden crystal galectin	1178	NM_001828	0.038
GPIHBP1	glycosylphosphatidylinositol anchored high density lipoprotein binding protein 1	338328	NM_178172	0.041
TSNARE1	t-SNARE domain containing 1	203062	NM_145003	0.042
FAM184A	family with sequence similarity 184, member A	79632	NM_001100411	0.043
ANKRD37	ankyrin repeat domain 37	353322	NM_181726	0.044
ODF3B	outer dense fiber of sperm tails 3B	440836	NM_001014440	0.044
PPP1R16B	protein phosphatase 1, regulatory subunit 16B	26051	NM_001172735	0.045
NIM1K	NIM1 serine/threonine protein kinase	167359	NM_153361	0.045
LYN	v-yes-1 Yamaguchi sarcoma viral related oncogene homolog	4067	NM_001111097	0.045
DNAJC3	DnaJ (Hsp40) homolog, subfamily C, member 3	5611	NM_006260	0.045
GFOD2	glucose-fructose oxidoreductase domain containing 2	81577	NM_001243650	0.045
C8orf58	chromosome 8 open reading frame 58	541565	NM_001013842	0.045
KCNA5	potassium voltage-gated channel, shaker-related subfamily, member 5	3741	NM_002234	0.046
SLCO4A1	solute carrier organic anion transporter family, member 4A1	28231	NM_016354	0.046
NTF4	neurotrophin 4	4909	NM_006179	0.046
PAK3	p21 protein (Cdc42/Rac)-activated kinase 3	5063	NM_001128166	0.048
EPB42	erythrocyte membrane protein band 4.2	2038	NM_000119	0.048
SLC44A3	solute carrier family 44, member 3	126969	NM_001114106	0.048
HPCAL1	hippocalcin-like 1	3241	NM_001258357	0.049
AOX1	aldehyde oxidase 1	316	NM_001159	0.049
MIF	macrophage migration inhibitory factor (glycosylation-inhibiting factor)	4282	NM_002415	0.049
PMEL	premelanosome protein	6490	NM_001200053	0.049
ARHGEF4	Rho guanine nucleotide exchange factor (GEF) 4	50649	NM_015320	0.049
PNCK	pregnancy up-regulated nonubiquitous CaM kinase	139728	NM_001039582	0.049
C5orf46	chromosome 5 open reading frame 46	389336	NM_206966	0.049
WDR60	WD repeat domain 60	55112	NM_018051	0.05

A ROC evaluation of the prediction accuracy was performed by plotting the sensitivity against 1– specificity for each result value of the test with tools available in BRBArray Tools. Three prediction algorithms were used to generate the ROC, including compound covariate predictor (CCP), diagonal linear discriminant analysis (DLDA), and Bayesian compound covariate predictor (BCCP). The analysis yielded a very modest but comparable ROC (Fig not shown) for all three algorithms with AUC of (0.226 (CCP), 0.246 (DLDA), 0.227 (BCCP)). Nonetheless, the association of 10 genes (LEP, HTRA4, SPAG4, LHB, TREM1, FSTL3, CGB, INHA, PROCR, and LTF) with PE placentae was highly consistent and significant at p < 0.001 (Table 3).

We further evaluated the currency of gene-to-publication ranks of these PPA genes by probing the scientific literature with GLAD4U (Gene List Automatically Derived For You, [34]). The search retrieved 6,288 publications, of which 642 contained information on 493 genes related to PE placenta. After ranking, 76 genes were significant (p<0.01) and prioritised as highly relevant to PE placenta (Table 4). The overlap between GLAD4U genes and PPA genes showed that only 6 of the latter genes (FLT1, ENG, INHA, LEP, PAPPA2, and HTRA4) were scored as significant and highly relevant from GLAD4U. Interestingly, 3 of these genes (LEP, HTRA4, INHA) also appeared in the top 10 of the PPA genes (Table 3). Similarly, fewer than expected GLAD4U genes (Table 5) were respectively identified in the PE Relative genes (22 genes), and PE Absolute genes (49 genes). Collectively, about 36% of genes identified from literature as highly relevant for PE placenta could not be confirmed as significant or consistently expressed in PE placentae following a large scale microarray meta-analysis.

**Table 4:** Highly Relevant Pre-eclamptic Placentae Genes from GLAD4U.

Rank	Gene ID	Gene Symbol	Species	Score
1	2321	FLT1	Homo sapiens	98.65391
2	5228	PGF	Homo sapiens	67.10936
3	2022	ENG	Homo sapiens	41.58958
4	29124	LGALS13	Homo sapiens	22.95614
5	8521	GCM1	Homo sapiens	21.84667
6	219736	STOX1	Homo sapiens	16.71948
7	3135	HLA-G	Homo sapiens	13.52779
8	6866	TAC3	Homo sapiens	9.593853
9	30816	ERVW-1	Homo sapiens	8.92392
10	3814	KISS1	Homo sapiens	7.901808
11	10761	PLAC1	Homo sapiens	7.26706
12	3491	CYR61	Homo sapiens	6.753044
13	7422	VEGFA	Homo sapiens	6.501511
14	283120	H19	Homo sapiens	6.323999
15	3091	HIF1A	Homo sapiens	5.995539
16	3291	HSD11B2	Homo sapiens	5.749669
17	3623	INHA	Homo sapiens	5.683964
18	133	ADM	Homo sapiens	5.396744
19	60676	PAPPA2	Homo sapiens	5.346197
20	5069	PAPPA	Homo sapiens	5.329039
21	406992	MIR210	Homo sapiens	5.306165
22	405754	ERVFRD-1	Homo sapiens	5.283019
23	4856	NOV	Homo sapiens	5.282213
24	94031	HTRA3	Homo sapiens	5.222811
25	666	BOK	Homo sapiens	5.16531
26	4973	OLR1	Homo sapiens	4.838615
27	1906	EDN1	Homo sapiens	4.30502
28	3791	KDR	Homo sapiens	4.239487
29	1647	GADD45A	Homo sapiens	4.229643
30	366	AQP9	Homo sapiens	4.218389
31	84432	PROK1	Homo sapiens	4.218389
32	203100	HTRA4	Homo sapiens	4.195596
33	285	ANGPT2	Homo sapiens	4.056039
34	1839	HBEGF	Homo sapiens	4.030243
35	7043	TGFB3	Homo sapiens	3.965444
36	3308	HSPA4	Homo sapiens	3.936441
37	308	ANXA5	Homo sapiens	3.913405
38	1506	CTRL	Homo sapiens	3.903702
39	4838	NODAL	Homo sapiens	3.871352
40	64073	C19ORF33	Homo sapiens	3.824958
41	11186	RASSF1	Homo sapiens	3.824184
42	185	AGTR1	Homo sapiens	3.818463
43	5806	PTX3	Homo sapiens	3.776246
44	3624	INHBA	Homo sapiens	3.62444
45	284	ANGPT1	Homo sapiens	3.395681
46	92	ACVR2A	Homo sapiens	3.378521
47	10887	PROKR1	Homo sapiens	3.326212
48	811	CALR	Homo sapiens	3.282796
49	3626	INHBC	Homo sapiens	3.073992
50	6338	SCNN1B	Homo sapiens	3.049242
51	10699	CORIN	Homo sapiens	2.931029
52	3952	LEP	Homo sapiens	2.826923
53	1E+08	PP13	Homo sapiens	2.819727
54	2689	GH2	Homo sapiens	2.808886
55	6870	TACR3	Homo sapiens	2.722579
56	719	C3AR1	Homo sapiens	2.722579
57	1392	CRH	Homo sapiens	2.688973
58	7020	TFAP2A	Homo sapiens	2.666521
59	6424	SFRP4	Homo sapiens	2.625932
60	186	AGTR2	Homo sapiens	2.527153
61	1491	CTH	Homo sapiens	2.476149
62	6510	SLC1A5	Homo sapiens	2.446266
63	3162	HMOX1	Homo sapiens	2.423731
64	4012	LNPEP	Homo sapiens	2.417434
65	284100	YWHAEP7	Homo sapiens	2.343263
66	391533	FLT1P1	Homo sapiens	2.343263
67	1096	CEACAMP8	Homo sapiens	2.343263
68	4543	MTNR1A	Homo sapiens	2.336584
69	6515	SLC2A3	Homo sapiens	2.298985
70	9166	EBAG9	Homo sapiens	2.206506
71	4318	MMP9	Homo sapiens	2.15965
72	80831	APOL5	Homo sapiens	2.122071
73	2153	F5	Homo sapiens	2.046305
74	442899	MIR325	Homo sapiens	2.043218
75	259	AMBP	Homo sapiens	2.039557
76	356	FASLG	Homo sapiens	2.034258

**Table 5:** Distribution of GLAD4U Genes in PE Gene Sets.

In PPA Genes	In Relative Genes	In Absolute Genes	
FLT1	FLT1	FLT1	ANXA5
ENG	ENG	PGF	NODAL
INHA	TAC3	ENG	AGTR1
PAPPA2	KISS1	LGALS13	PROKR1
HTRA4	PLAC1	GCM1	CALR
LEP	VEGFA	STOX1	INHBC
	INHA	HLA-G	SCNN1B
	PAPPA2	TAC3	LEP
	KDR	ERVW-1	GH2
	HTRA4	KISS1	TACR3
	ANXA5	PLAC1	CRH
	AGTR1	CYR61	TFAP2A
	INHBA	HSD11B2	SFRP4
	ANGPT1	INHA	AGTR2
	CALR	ADM	CTH
	SCNN1B	PAPPA2	SLC1A5
	LEP	PAPPA	HMOX1
	C3AR1	ERVFRD-1	LNPEP
	CRH	NOV	MTNR1A
	TFAP2A	HTRA3	SLC2A3
	SLC2A3	OLR1	APOL5
	F5	EDN1	F5
		HTRA4	AMBP
		ANGPT2	FASLG
		TGFB3	

### Biological Relevance of the Significant Genes

Using the AGE results, we examined the biological relevance, the similarities and differences between the NP and PE placental gene sets. KEGG pathway maps were probed with WebGestalt (WEB-based GEne SeT AnaLysis Toolkit). Altogether, 207 and 126 KEGG pathways (S8 & S9 Tables) were significantly enriched respectively by PE and NP placental Absolutes genes (p<0.05, with Hypergeometric tests: multiple testing correction (MTC) and BH; and a minimum gene threshold of 2). All 126 pathways enriched by the NP placental genes were also affected significantly in PE, but with higher enrichment ratios. We observed additional 81 pathways that were significantly affected only in PE placentae. The most highly affected pathways in PE include: Wnt signaling pathway; Long-term potentiation; Melanoma; TGF-beta signaling pathway; T cell receptor signaling pathway; ErbB signaling pathway; mRNA surveillance pathway; PPAR signaling pathway; Ubiquitin mediated proteolysis; and Hedgehog signaling pathway (Table 6).

**Table 6:** List of KEGG Pathways identified exclusively in PE Placenta.

Pathway Name	
Wnt signaling pathway	Intestinal immune network for IgA production
Long-term potentiation	Valine, leucine and isoleucine biosynthesis
Melanoma	One carbon pool by folate
TGF-beta signaling pathway	Renin-angiotensin system
T cell receptor signaling pathway	Alanine, aspartate and glutamate metabolism
ErbB signaling pathway	African trypanosomiasis
mRNA surveillance pathway	Fructose and mannose metabolism
PPAR signaling pathway	Dorso-ventral axis formation
Ubiquitin mediated proteolysis	Thyroid cancer
Hedgehog signaling pathway	NOD-like receptor signaling pathway
Asthma	Glycosaminoglycan biosynthesis—heparan sulfate
Valine, leucine and isoleucine degradation	Nicotinate and nicotinamide metabolism
Pyrimidine metabolism	Inositol phosphate metabolism
Type II diabetes mellitus	Mucin type O-Glycan biosynthesis
Butanoate metabolism	Other glycan degradation
RNA degradation	Bladder cancer
Ribosome biogenesis in eukaryotes	Nitrogen metabolism
Primary immunodeficiency	Glycerophospholipid metabolism
Progesterone-mediated oocyte maturation	Synthesis and degradation of ketone bodies
Amyotrophic lateral sclerosis (ALS)	Glycosphingolipid biosynthesis—globo series
Glycosphingolipid biosynthesis—lacto and neolacto series	RNA polymerase
Carbohydrate digestion and absorption	SNARE interactions in vesicular transport
Galactose metabolism	Primary bile acid biosynthesis
Fc epsilon RI signaling pathway	Basal transcription factors
Propanoate metabolism	Glycosaminoglycan biosynthesis—chondroitin sulfate
Allograft rejection	Glycosylphosphatidylinositol(GPI)-anchor biosynthesis
Apoptosis	Nucleotide excision repair
Endometrial cancer	Glycosaminoglycan biosynthesis—keratan sulfate
Peroxisome	Biosynthesis of unsaturated fatty acids
VEGF signaling pathway	Phenylalanine, tyrosine and tryptophan biosynthesis
Histidine metabolism	Circadian rhythm—mammal
p53 signaling pathway	Pantothenate and CoA biosynthesis
Non-small cell lung cancer	Glycosaminoglycan degradation
Type I diabetes mellitus	Glycine, serine and threonine metabolism
Fatty acid metabolism	Fatty acid elongation in mitochondria
Glyoxylate and dicarboxylate metabolism	DNA replication
Basal cell carcinoma	Base excision repair
Graft-versus-host disease	Folate biosynthesis
Tyrosine metabolism	D-Glutamine and D-glutamate metabolism
Lysine degradation	Ether lipid metabolism
Glycerolipid metabolism	

Pathways in order of descending adjusted significance levels (details of pathways available in **S8** Table).

We repeated the analysis with the Relative significant genes, and 176 pathways were significantly affected in PE placentae. Of these, 164 were correctly mapped to Absolute genes affected pathways, but with variations in enrichment ratios. Examination of the 12 pathways affected only by the Relative Genes showed closer links with metabolism: (ID: Mismatch repair; Homologous recombination; Selenocompound metabolism; Sulfur relay system; Steroid biosynthesis; Terpenoid backbone biosynthesis; Biotin metabolism; Vitamin B6 metabolism; Fatty acid biosynthesis; Riboflavin metabolism; Ubiquinone and other terpenoid-quinone biosynthesis; Caffeine metabolism).

## Discussion

PE is a serious complication of human pregnancy. While previous studies have led to clear descriptions of symptoms and diagnosis, our understanding of the genes altered in PE is still limited. In an attempt to identify a common set of dysregulated genes in PE placentae, we subjected a thoroughly screened subset of existing datasets to a robust set of analyses. Interestingly, the data revealed that over a third of the genes identified in the literature as being implicated in PE, were not identified as associated with or consistently expressed in PE placentae. This raises the question of whether current trends in PE genomic investigations are accurately reflecting the true nature of the molecular pathology of the condition.

In cognisance of this, we identified specific gene sets that have not been previously reported for PE. Of these, there was an expectation that all the significant RGE genes would be mapped to the AGE PE genes. Rather, only 51% of the RGE genes were identified from the AGE PE genes. The remaining RGE significant genes showed varied levels of expression between PE and NP placentae but were classified as inconsistent with RankProd analysis. In contrast, 77% of the AGE genes did not match with the RGE genes. Of these, about 80% were genes that showed low or similar levels of expression in both PE and NP but were consistently expressed in PE placentae only.

Thus, the current findings show that the use of AGE analysis enables the description of a comprehensive, globally and consistently expressed PE placental genes. On the other hand, the findings show that overt use of RGE analysis to the disadvantage of AGE could limit gene sets and our understanding of the real time and complexities of changes that could occur in the PE state. These findings appear to confirm earlier reports [23,24] that RGE not only identifies limited candidate genes but could also exclude large proportion of genes that may be of relevance in characterising the molecular pathology of a disease including those with low level expression and genes with similar levels of expression in both the case and control samples. The findings also seem to suggest that RGE could inherently identify genes whose expression patterns may be inconsistent but might have large differential expression between control and case samples.

Generally, the roles of genes with low level expressions in a disease state are unclear. However, reports from stem cell research suggest that low level gene expression may be involved in lineage priming and cell differentiation [35–38]. While such conclusions cannot be inferred as yet in the placenta from the current study, our findings showed that the PE placenta retains its ability to express the genes significantly regulated in NP placenta. The findings also showed the presence of additional subsets of unique genes including low level expressed genes that were consistently expressed only in PE placentae.

It could thus, be inferred from the current findings, albeit limited to RNA messages that: (1) there may be apparent expression of a set of genes, that could be critical for the survival or development of the placenta, and the pattern of expression of these genes might be similar in both NP and PE placentae; (2) in PE placentae, there may be consistent regulation of excess pool of genes (PE unique genes), that may exacerbate the activation of pregnancy-favourable biological pathways or precipitate pregnancy-unfavourable biological pathways; (3) PE may be a polygenic condition decompensated by the cumulative effect of multiple genes, each with small effects, and there may be no single gene with a large effect. These were most evident in the extent to which the molecular interaction and reaction pathways were affected in the PE placentae.

We identified two sets of pathways: common pathways in both NP and PE placentae, and unique pathways affected only in PE. The observation that the common pathways were enriched either more negatively or positively in PE than in NP appeared to suggest a plausible decompensation or exaggeration of normal placental functions as key factors in PE. Perhaps, of greatest significance for future research is the identification of previously unidentified dysregulated pathways in PE placentae such as: Histidine metabolism, Fc epsilon RI signaling pathway, allograft rejection, graft vs. host disease, primary immunodeficiency and renin-angiotensin, Wnt signaling, RNA degradation, and RNA Polymerase.

Wnt signaling, RNA degradation, and RNA Polymerase pathways were significantly affected only in PE. The canonical Wnt pathway leads to regulation of gene transcription [39], suggesting that PE could be linked to excessive gene expression in response to an autacoids or a paracrine hormones such as histamine with regulatory roles on Wnt pathway [40].

Crucially, dysregulated metabolism of histamine as a consequence of impaired histidine metabolism in pregnancy is well known to affect PE [41,42]. Therefore, the concurrent identification of Histidine metabolism pathway in PE is of significance. Possibly, the cumulative effects of the release of the histamine and other substances involved in inflammation and immune responses, cell proliferation, tissue differentiation, tumour formation, apoptosis and production of purines and pyrimidines is of importance [43,44]. Significantly, dysregulation of these functions are widely accepted to be rooted in the defects in early trophoblast to uterine invasion, adaptive transformation of the uterine spiral arteries to high capacity and low impedance vessels, and development of chorionic villi [14,45,46]. These are important issues known to affect PE, commonly at the early stages of the disease development [47].

Fc epsilon RI-mediated signaling pathway was also affected only in PE. This pathway in mast cells are initiated by interaction of multivalent allogens with the extracellular domain of the alpha chain of Fc epsilon RI to release preformed histamines, proteoglycans (especially heparin), phospholipase A2 and subsequently, leukotrienes (LTC4, LTD4 and LTE4), prostaglandins (especially PDG2), and cytokines including TNF-alpha, IL-4 and IL-5 [48]. These mediators and cytokines contribute to inflammatory responses.

In the case of inflammatory pathways in PE, it is suggested that the nuclear factor kappa-light-chain-enhancer of activated B cells (NF-κB) pathway mediates excessive maternal intravascular inflammation that leads to endothelial dysfunction [49,50]. In this context, it has been hypothesised that PE arises as a result of an excessive maternal intravascular inflammatory response to pregnancy, and that it involves the activation of both innate and the adaptive immune system, neutrophil, and the complement system pathways [50–54].

Similarly, we identified allograft rejection, graft vs. host disease, and primary immunodeficiency pathways as affected in PE. This observation is consistent with previous opinions that heightened immune responses in PE pregnancies could be a consequence of chronic feto-allograft rejection reaction [55]. Accordingly, PE shares similarities with graft rejection linked to over activation of immune pathways [56–61]. Integral to this is the argument that disequilibrium of Th1/Th2 cytokine balance in favour of Th1 (IL-2, IL-12, IL-15, IL-18, IFNgamma, TNFalpha vs. IL-4, IL-10, TGFbeta); precipitation of subsets of immunocompetent cells (T CD4, suppressor gammadeltaT, cytotoxic T CD8, Treg, Tr1, uterine NK cells); innate immunity (NK cytotoxic cells, macrophages, neutrophils and complement); adhesion molecules; fgl2 prothrombinase activation [56–61] and under-expression of Heme oxygenase-1 (HO-1) [62] underpin the development of PE.

This opinion is however not universally supported. A recent review by Ahmed and Ramma [63] appears to down-play the roles of inflammatory, hypoxia and immunologic pathways in favour of angiogenic response as the cause of PE. They argue that recent work supports the hypothesis that PE arises because of the loss of vascular endothelial growth factor (VEGF) activity, which in turn is caused by increase in the levels of endogenous soluble fms-like tyrosine kinase-1 (sFlt-1), an anti-angiogenic factor [63]. SFlt-1 binds and reduces free circulating levels of the pro-angiogenic factor VEGF, and thus inhibits the beneficial effects mediated by flt-1 (also known as vascular endothelial growth factor receptor 1 (VEGFR-1)) on maternal endothelium, with consequent maternal hypertension and proteinuria [64,65]. It is further argued that altered balance of circulating pro-angiogenic/anti-angiogenic factors such sFlt-1, soluble endoglin, and placenta growth factor (PlGF) are unique to PE [63–67]. This view is not lost as we also identified VEGF signaling pathway as affected only in PE.

However, due to the complexity of pathways affected in PE, our findings contrast the conclusions drawn by Ahmed and Ramma [63]. Instead, our findings support a more global view that multiple and concurrent dysregulated pathways underpin the aetiology of PE [47], and no single pathway could be associated with the origins of PE.

These findings therefore provide the opportunity to re-examine current studies in PE to reflect the consistently expressed genes that are unique to PE placentae or biological pathways, especially those that may be exclusively affected in PE placentae, to improve our understanding of the molecular pathology or the genomic basis of PE.

## Supporting Information

S1 Table Profile of Microarray Series Excluded from PE Meta-analysis. (XLSX)

S2 Table Profile of Samples Rejected after INMEX Quality Appraisal. (XLSX)

S3 Table Relative PE Genes (Expressions in PE Placenta relative to NP Placentae). (XLSX)

S4 Table Genes down-regulated in both NP and PE placentae. (XLSX)

S5 Table Genes exclusively down-regulated in PE. (XLSX)

S6 Table Genes Up-regulated in both PE and NP. (XLSX)

S7 Table Genes exclusively Up-regulated in PE (not in NP). (XLSX)

S8 Table Significantly Pathways Affected in PE Placentae. (XLSX)

S9 Table Significantly Affected Pathways in NP Placentae. (XLSX)

S10 Table PRISMA Checklist. (DOC)

S11 Table Full Electronic Search Strategy for Gene Expression Omnibus (GEO). (DOCX)
