# Supplementary material for: Comparison of Normal and Pre-Eclamptic Placental Gene Expression: A Systematic Review with Meta-Analysis
Source: PLoS One. 2016 Aug 25;11(8):e0161504. doi: 10.1371/journal.pone.0161504 (PMC4999138; doi:10.1371/journal.pone.0161504)
Supplement: S11 Table — (DOCX) [file pone.0161504.s011.docx]

**S11 Table: Full Electronic Search Strategy for Gene Expression Omnibus (GEO)**

| #1 | GEO Datasets |
| --- | --- |
| #2 | ("placenta"[MeSH Terms] OR placenta[All Fields]) AND ("pre-eclampsia"[MeSH Terms] OR preeclampsia[All Fields]) AND ("gse"[Filter] AND "Homo sapiens"[Organism] AND "Expression profiling by array"[Filter]) |
